# Enhancing RBD exposure and S1 shedding by an extremely conserved SARS-CoV-2 NTD epitope

**DOI:** 10.1038/s41392-024-01940-y

**Published:** 2024-08-28

**Authors:** Qianhui Zhu, Pan Liu, Shuo Liu, Can Yue, Xiangxi Wang

**Affiliations:** 1 CAS Key Laboratory of Infection and Immunity, National Laboratory of Macromolecules, Institute of Biophysics, Chinese Academy of Sciences, Beijing, 100101 China; 2 Changping Laboratory, Beijing, China

**Keywords:** Structural biology, Molecular biology

**Dear Editor,**

Multiple waves of outbreaks of severe acute respiratory syndrome coronavirus 2 (SARS-CoV-2) have resulted in unprecedented public health and socioeconomic crises. After 4 years of primary as well as breakthrough infections and under the increased immune pressure exerted by vaccination, SARS-CoV-2 has evolved into multiple variants that displayed either enhanced transmissibility or immune escape properties. The currently dominant variant, BA.2.86 sublineages, with over 35 mutations in Spike (S), showed higher immune evasion and formed a distinct BA.2.86 sub-lineage branch via phylogenetic analysis of the primary sequences of S.^1^ To mitigate the spread of epidemic and impact on public health, a large number of monoclonal antibodies have been developed and deployed rapidly. The class I anti-RBD NAbs like DXP-604 and LY-CoV016, which bind RBD “up” conformation, can block ACE2 binding and have strong neutralizing activity.^2^ However, RBD is also a domain with a very high mutation frequency, rendering most clinically authorized anti-RBD NAbs ineffective against new BA.2.86 sublineages harboring such mutations.^3^ Among the four classes of anti-NTD NAbs (α, β, γ, δ),^2^ the first three classes antibodies have been evaded due to antigenic changes. Here, we found that two δ-class antibodies, XG2v046 and XGv280, which recognize the conserved epitope of NTD and can enhance RBD exposure and S1 shedding by promoting RBD to assume “up” conformation as a neutralizing mechanism, have broad-spectrum neutralizing effects on SARS-CoV-2 variants. Importantly, the new regulatory mechanism of the anti-NTD NAbs enables those anti-“up” RBD NAbs that were nearly ineffective against new variants to regain effectiveness and broaden their spectrum of activity. As such, the host immune system has developed an antiviral mechanism against ongoing antigenic variation via a secondary-protective barrier formed by a subset of conserved anti-NTD antibodies represented by XG2v046 and XGv280 in synergy with partial anti-RBD antibodies.

We conducted pseudovirus neutralization assay for various variants of SARS-CoV-2 using antibodies isolated from volunteers. The results showed that most anti-RBD and anti-NTD antibodies were ineffective against different variants (Fig. 1a). Interestingly, two NTD antibodies, XG2v046 and XGv280, neutralized the full spectrum of SARS-CoV-2 variants ranging from the earliest D614G to the more recent JN.1 with medium potency (Fig. 1a). In order to identify the epitopes, we determined the structures of BA.2.86 S-trimer in complex with Fab fragments of XG2v046 and XGv280 at 3.40 Å and 3.75 Å resolution, respectively (Fig. 1b). Both Abs bind to “right shoulder” of NTD, like XG2v024^2^ from the “δ” class of NTD antibodies. XG2v046, XGv280 and XG2v024 share 6 identical residues (Y28, P85, N87, T108, T109 and R237) involved in NTD interactions. The epitopes of the two anti-NTD NAbs are conserved among VOC, VOI of SARS-CoV-2, and even RaTG13. These analyses of sequences and structures analy are consistent with binding affinities of XG2v046 and XGv280 to RaTG13 S-trimer, explaining the ultra-broad neutralization breadth.

**Fig. 1:**
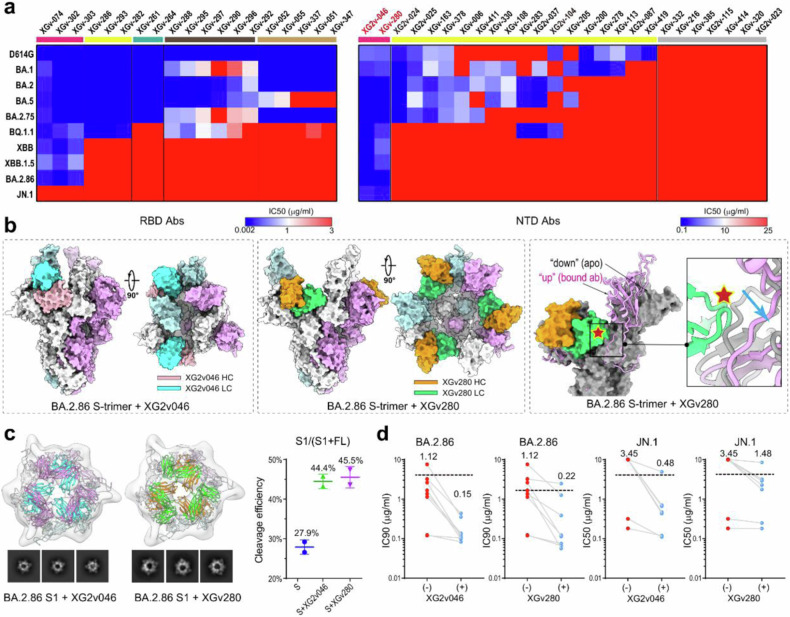
Functional and structural characterization of antibodies. **a** Heatmap of neutralizing activity of representative anti-RBD (left) and anti-NTD (right) antibodies against D614G, BA.1, BA.2, BA.5, BA.2.75, BQ.1.1, XBB, XBB.1.5, BA.2.86 and JN.1. **b** Cryo-EM structure of BA.2.86 S-trimer in complex with XG2v046 or XGv280. Surface representation of S-trimer in complex with XG2v046 and XGv280 was shown on the left, while steric clash between XGv280 light chain and *apo* BA.2.86 down RBD was shown on the right. **c** Neutralization mechanism of the two NTD NAbs. Density maps and 2D classes of BA.2.86 S1 subunit in complex with XG2v046 or XGv280 were shown on the left, while Western blot analysis of BA.2.86 S-trimer protein, and its mixture with XG2v046 or XGv280 probed with antibodies against S1, with quantification of the ratio of S1 to total spike was shown on the right. Data are mean ± SEM of 2 biological replicates. **d** Enhancement of neutralization potency against BA.2.86 and JN.1 for NAbs bound to up RBD conformation. The dash line indicated the neutralization potency of XG2v046 or XGv280. Geometric means of IC90 or IC50 for each group of antibodies were marked

However, unlike XG2v024, which locks S in a closed conformation,^2^ both XG2v046 and XGv280 placed RBD in “up” conformation, with the binding of XGv280 triggering the opening of RBD at a larger angle compared to XG2v046 (Fig. 1b). Such conformational transitions were distantly regulated by binding sites on NTD, rather than masking fusion peptide and did not interfere with recognition of hACE2. By comparing the RBD “down” and “up” conformations of *apo* BA.2.86 S-trimer with the RBD “up” conformation triggered by XGv280 binding, we found that the counterclock-wise rotation of RBD (from down to up) was accompanied by the counter clock-wise rotation of NTD, which indicated that the moving tracks for RBD and NTD were inversely correlated, where RBD tended to shift upwards and NTD inclined to move downwards. Binding of XGv280 to NTD pushes the linker (residues 528–532, between RBD and subdomain 1) back-downward by up to 7 Å, allosterically leading to a more erected RBD and thereby conferring loose and flexible upper architecture formed by three S1 subunits (Fig. 1b). Perhaps correlated with this, we observed low-resolution structures of the dissociated S1 trimer in complex with three copies of XG2v046 or XGv280 Fabs (Fig. 1c). To further verify this, we compared the spike cleavage efficiencies with or without treatments of XG2v046/XGv280 and observed dramatically improved cleavage in spike when treated by XG2v046/XGv280 as evidenced by the ratio of full-length spike to S1 (Fig. 1c). These results unveil a neutralization mechanism for XGv280 and XG2v046 by facilitating spike cleavage and early S1 shedding via allosteric regulation. Although XG2v046 and XGv280 bind to conserved epitopes on NTD, the ratio of RBD “down” versus “up” for the *apo*-state of JN.1 S is higher than that in BA.2.86.^1^ So, shedding of S1 subunit was more difficult for JN.1 than BA.2.86, which can explain the decrease in neutralization activity of XG2v046 and XGv280 against JN.1 compared to BA.2.86. (Fig. 1a).

The coronavirus S-trimer can be maintained in an open conformation via multiple mechanisms like NTD binding to sialic acid.^4^ Surprisingly, XG2v046 and XGv280 exert a novel allosteric regulatory mechanism (Fig. 1b). In fact, antibodies targeting “γ” epitopes on NTD, such as 8D2, can also induce the open conformation of RBD to enhance infectivity of SARS-CoV-2 in vitro.^5^ However, the changes in the antigenic sites of variants after BA.1 in NTD results in a direct loss of binding ability.^2^ Theoretically, the “RBD up” state is more conducive to binding antibodies targeting RBM region from class I-IV. In order to test this hypothesis, we selected a bunch of anti-RBD antibodies to test the neutralization potency against BA.2.86 and JN.1 with or without XG2v046 or XGv280. These anti-RBD antibodies meet the two requirements: (1) only bind to RBD “up” conformation; (2) only have weak neutralization potency (IC90 > 0.1 μg/ml). Compared with antibodies which were not mixed with XG2v046, the neutralization potency of the mixture (anti-RBD NAb mixed with XG2v046 at a ratio of 1:1) against BA.2.86 (IC90) or JN.1 (IC50) increased 7.5-fold or 7.2-fold on average, respectively (Fig. 1d). A similar trend was observed for XGv280, but the neutralization potency was enhanced to a smaller extent (5.5-fold for BA.2.86 and 2.3-fold for JN.1) (Fig. 1d). However, such synergistic effects were not observed when cocktailed with sotrovimab (S309) since that S309 can bind to S-trimer either in “RBD up” or “RBD-down” conformation. Thus, a small number of antibodies binding the “δ” class epitope found on NTD could partially recover neutralizing activities of some weakly neutralizing anti-RBD NAbs binding to “RBD up” conformation. Our results indicate that a subset of anti-NTD antibodies represented by XG2v046 and XGv280 in combination with partial anti-RBD antibodies form a secondary-protective barrier in ongoing antigenic variation driven by selective pressure, revealing a new antiviral mechanism evolved by host immune system.

### Supplementary information

Enhancing RBD exposure and S1 shedding by an extremely conserved SARS-CoV-2 NTD epitope

## Data Availability

The atomic coordinates of BA.2.86 S-trimer in complex with XG2v046 and XGv280 have been deposited in the Protein Data Bank (PDB) under accession codes 8Y4A and 8Y4C, respectively. Cryo-EM density maps of BA.2.86 S-trimer in complex with XG2v046 and XGv280, have been deposited at the Electron Microscopy Data Bank with accession codes EMD-38914 and EMD-38915.
