# Supplementary material for: Enhancing RBD exposure and S1 shedding by an extremely conserved SARS-CoV-2 NTD epitope
Source: Signal Transduct Target Ther. 2024 Aug 28;9:217. doi: 10.1038/s41392-024-01940-y (PMC11349971; doi:10.1038/s41392-024-01940-y)
Supplement: Supplementary file 1 — Enhancing RBD exposure and S1 shedding by an extremely conserved SARS-CoV-2 NTD epitope [file 41392_2024_1940_MOESM1_ESM.docx]

Supplementary Materials for

**Enhancing RBD exposure and S1 shedding by an extremely conserved NTD epitope**

**Authors:** Qianhui Zhu^1$^, Pan Liu^1$^, Shuo Liu^2$^, Can Yue^1^* and Xiangxi Wang^1,2^*

^1^ CAS Key Laboratory of Infection and Immunity, National Laboratory of Macromolecules, Institute of Biophysics, Chinese Academy of Sciences, Beijing 100101, China

^2^ Changping Laboratory, Beijing

**This PDF file includes:**

Methods and Materials

**Supplementary information**

**Antibody clustering**

For t-SNE analysis of RBD antibodies, each antibody was represented as a vector according to the site DMS scores published in previous research^1^. These D-dimensional representations were further embedded into two-dimensional space for visualization with t-SNE using sklearn.manifold.TSNE of scikit-learn. For t-SNE analysis of NTD antibodies, due to the shortage of DMS scores, we represented these antibodies according to the epitopes instead. In other words, we collected 29 structures of SARS-CoV-2 NTDs in complex with antibodies from PDB website and the residues within 4 Å from CDRs were identified as epitopes. Each NTD antibody was represented by a vector where the epitope is represented by 1 and otherwise by zero. Package of sklearn.manifold.TSNE of scikit-learn was also used to dimensionality reduction.

**Antibody expression and Fab generation**

The target genes of antibodies were codon-optimized and constructed on plasmids encoding human IgG1 Fc as described previously. The light and heavy chain plasmids of the antibodies were then transiently transfected into mammalian HEK 293F cells at 1:1 ratio. The mammalian HEK 293F cells were incubated for 5 days in a 5% CO2 rotating incubator set at 37 °C for antibody expression. Then, the cell supernatant was harvested and further purified with protein A beads. Finally, the antibody was exchanged into phosphate buffered saline (PBS). To generate the Fab fragments, the purified NAbs were processed using the Pierce FAB preparation kit (ThermoScientific) as described previously. In brief, the samples were first applied to desalination columns to remove the salt and the flow-throughs were collected and incubated with papain that was attached with beads to cleave Fab fragments from the whole antibodies for 5 h at 37 °C. After that, the mixtures were transferred into protein A columns and the flow-throughs, that is, the Fab fragments were collected and dialyzed into PBS (ThermoFisher, catalogue (cat.) no. 10010023).

**Pseudovirus neutralization assay**

The pseudotyped viruses bearing the S protein were generated, aliquoted and restored as previously described^2^. In brief, 293T cells were first transfected with the plasmid embedded with the S gene of D614G or variants (BA.1, BA.2, BA.5, BA.2.75, BQ.1.1, XBB, XBB.1.5, BA.2.86 and JN.1) of SARS-CoV-2. The transfected 293T cells were infected with VSV G pseudotyped virus (G*ΔG-VSV) at a multiplicity of infection (MOI) of 4. After incubation for 5 h, cells were washed with PBS, and then complete culture medium was added. After another 24 h, the SARS-CoV-2 pseudoviruses were produced and collected. For the *in vitro* pseudotyped virus neutralization assay, the antibodies were diluted in DMEM starting from 10 μg/ml with 6 additional threefold serial dilutions, each of which were mixed with the collected pseudovirus and incubated at 37 °C for 1 h.

For the synergistic pseudovirus neutralization assay of anti-RBD antibodies and anti-NTD antibodies, we mixed anti-RBD antibodies and anti-NTD antibodies in an equal ratio of 1:1, and then adjusted the final concentration after mixing to 10 μg/ml. Thus, the final concentrations of anti-RBD antibodies and anti-NTD antibodies were 5 μg/ml, respectively. Three-fold serial dilutions of the antibodies were made in DMEM, mixed with the collected pseudovirus and incubated at 37°C for 1 h.After that, the mixtures were added to Huh-7 cells and placed back for incubation for another 24 h. Then, the luciferase luminescence (RLU) of each well was measured with a luminescence microplate reader. The neutralization percentage was calculated as following: Inhibition (%) = (1 − (sample RLU − blank RLU) / (positive control RLU − blank RLU)). Antibody neutralization titers were presented as 50% or 90% maximal inhibitory concentration (IC50/IC90).

**Protein expression and purification**

The sequence of BA.2.86 full-length S protein (residues 1–1208) was modified from the plasmids encoding the S and RBD of WT SARS-COV-2 (GenBank: MN908947) in our lab by overlapping PCR. The proline substitutions at 817, 892, 899, 942, 986 and 987, ‘GSAS’ substitutions at the S1/S2 furin cleavage site (residues 682–685) and a C-terminal T4 foldon trimerization domain were also introduced in the BA.2.86 S construct to stabilize the trimeric conformation of S protein. For protein expression, the plasmids of these proteins were transiently transfected into HEK 293F cells grown in suspension at 37 °C in an incubator supplied with 8% CO2, rotating at 130 rpm. The cell supernatants were collected and concentrated three days post-transfection, and further purified by affinity chromatography using resin attached with streptavidin and size-exclusion chromatography (SEC) using a Superdex 6 10/300 column (GE Healthcare Life Sciences) equilibrated with the buffer containing PBS.

**Bio-layer interferometry**

BLI experiments were run on an Octet Red 96 instrument (Fortebio). Monoclonal antibodies were immobilized onto Protein A biosensors (Fortebio) and double serial dilutions of Spike (RaTG13) in PBS were used as analytes. To measure the binding affinities of S-ECD-His or mixture (molar ratio of S-ECD-His and anti-NTD antibodies, 1:10) with fusion peptide antibody (76E1)^3^, S-ECD-His or mixture (S-ECD-His and anti-NTD antibodies) were immobilized on NTA biosensors (Fortebio) and threefold serial dilutions of 76E1 in PBS were used as analytes. Data were then analyzed using software Octet BLI Analysis 9.0 (Fortebio) with a 1:1 fitting model. For the competitive assay, SARS-CoV-2 S-ECD-His tagged with His (Sino Biological, 40589-V08B1) was immobilized on NTA biosensors, which were pre-equilibrated in the buffer for at least 1 min. The immobilized biosensors were immersed with the first monoclonal antibody (XG2v046 or XGv280) for 300 s, followed by addition of the second monoclonal antibody (76E1) for another 300 s. Data obtained were also analysed by Octet BLI Analsis 9.0.

**Surface plasmon resonance (SPR)**

SPR experiments were performed on the Biacore 8K (GE Healthcare). For the binding affinity assays, purified hACE2 (His tag) was immobilized onto a CM5 sensor chip surface using the NHS/EDC method and a PBS running buffer (supplemented with 0.05% Tween-20). Double serial dilutions of BA.2.86-S trimer (His and Strep II tags) or mixture (molar ratio of BA.2.86-S trimer and antibodies, 1:10) were injected. Among them, the RBD antibody (BD55-1205)^4^ binds to the RBD "up "conformation and binds to the RBM region. The response units (RU) were recorded by Biacore 8K Evaluation Software (GE Healthcare) at room temperature, and the raw data curves were fitted to a 1:1 binding model using Biacore 8K Evaluation Software (GE Healthcare).

**Western blot analysis**

In order to verify the effect of anti-NTD antibodies on enhancing S1 shedding, western blotting experiments were performed. First, we incubated BA.2.86-S trimer and anti-NTD antibodies (XG2v046 and XGv280), binding but not neutralizing against BA.2.86) at 4 ℃ for 2 ~ 3h at a molar ratio of 1:10, respectively. Then, Trypsin with final concentration of 1 ug/ml was added into BA.2.86-S trimer and mixture (BA.2.86-S trimer with antibodies), and incubated at 37 ℃ for 15min. Next, PMSF with a final concentration of 1mM was added, Trypsin was terminated, and incubated on ice for 10min. Then, the sample was diluted with 5 × SDS loading buffer and boiled for 10min. Finally, the sample size of each well contained 100 ng BA.2.86-S trimer protein for western blotting analysis. For protein detection, the following antibodies were used: mouse anti-SARS-CoV-2 S1 antibodies (Sino Biological, 40592-MM117), mouse anti-SARS-CoV-2 S2 antibodies (GeneTex, GTX632604), horseradish peroxidase (HRP)-conjugated anti-mouse IgG polyclonal antibodies (Thermo Fisher Scientific, A16078). Chemiluminescence was detected using the ChemiDoc Touch Imaging System (Bio-Rad). The cleavage ratio of S1 or S2 to FL in virions was determined by densitometry using ImageJ (NIH).

**Cryo-EM sample preparation and data collection**

The purified BA.2.86 S protein was mixed separately with the Fab fragments of XGv302+XG2v046 and XGv280 at a molar ratio of 1: 1.2 and kept on ice for 10 s. After that they were dropped onto the pre-glow-discharged holey carbon-coated gold grid (C-flat, 300-mesh, 1.2/1.3, Protochips In.), blotted for 6 s with no force in 100% relative humidity and immediately plunged into the liquid ethane using Vitrobot (FEI). Cryo-EM data sets of these complexes were collected at 300 kV with an FEI Titan Krios microscope (FEI). Movies (32 frames, each 0.2 s, total dose of 60 e− Å−2) were recorded using a K2 or K3 Summit direct detector with a defocus range between 1.2–2.0 μm. Automated single particle data acquisition was carried out by Serial EM, with a calibrated magnification of 22,500 yielding a final pixel size of 1.04 or 1.07 Å.

**Cryo-EM data processing**

A total of 5,187 and 2,167 micrographs of S–XGv302+XG2v046 complex and S–XGv280 complex, respectively, were recorded and subjected to beam-induced motion correction using motionCorr2 in RELON3.0 package. The defocus values of micrographs were calculated by Gctf. Then, 2,494,422 and 853,116 particles of S–XGv302+XG2v046 complex and S–XGv280 complex, respectively, were picked and extracted for reference-free 2D alignment by cryoSPARC. 982,274 and 365,237 particles were selected and applied for Ab-Initio Reconstruction for S–XGv302+XG2v046 complex and S–XGv280 complex, respectively, with no symmetry imposed to produce the potential conformations for the complexes. After that, the candidate model for each complex was selected and processed by auto-refine and postprocessing in cryoSPARC to generate the final cryo-EM density for S–XGv302+XG2v046 complex and S–XGv280 complex. The resolution was determined by gold-standard Fourier shell correlation (threshold = 0.143) and evaluated by ResMap.

**Model fitting and refinement**

The atomic models of S-XG2v046 complex and S–XGv280 complex were generated by first fitting the chains of the native *apo* SARS-CoV-2 S trimer (PDB number: 6VYB) and XG2v024 Fab (PDB number: 7YR1) into the cryo-EM densities of the final S-Fab-complexes described above by Chimera, followed by manual adjustments and corrections according to the protein sequences and densities in Coot, as well as real space refinement using Phenix.

**Reference**

1. Cao, Y et al. BA.2.12.1, BA.4 and BA.5 escape antibodies elicited by Omicron infection. *Nature*. **608**: 593-602 (2022).
2. Nie, J et al. Establishment and validation of a pseudovirus neutralization assay for SARS-CoV-2. *Emerg Microbes Infect*. **9**: 680-686 (2020).
3. Sun, X et al. Neutralization mechanism of a human antibody with pan-coronavirus reactivity including SARS-CoV-2. *Nature Microbiology*. **7**: 1063-1074 (2022).
4. Jian, F et al. A generalized framework to identify SARS-CoV-2 broadly neutralizing antibodies. *bioRxiv*. <https://doi.org/10.1101/2024.04.16.589454> (2024).
